# Organic Amendment Quality Regulates Greenhouse Gas Trade-Offs and Short-Term Carbon Retention During Reductive Soil Disinfestation

**DOI:** 10.3390/metabo16090653

**Published:** 2026-09-06

**Authors:** Shanju Wen, Shijuan Xiong, Weimo Wu, Jinhu Zhi, Weiyang Liu, Chunming Chi, Lu Kang, Xiaohong Tian

**Affiliations:** 1 College of Agriculture, Tarim University, Alar 843300, China; 120050106@taru.edu.cn (S.W.);; 2 College of Natural Resources and Environment, Northwest Agriculture and Forestry University, Yangling, Xianyang 712100, China; 3 Institute of Pepper, Guizhou Academy of Agricultural Sciences, Guiyang 550009, China; 4 Institute of Agricultural Quality Standards and Testing Technology, Xinjiang Academy of Agricultural Sciences, Urumqi 830091, China

**Keywords:** reductive soil disinfestation, organic amendment C/N ratio, soil organic carbon fractions, soil enzyme activities, microbial carbon mineralization, greenhouse gas emissions

## Abstract

**Highlights:**

**What are the main findings?**

- Low-C/N wheat straw promoted enzyme-driven C mineralization and increased SOC but stimulated methanogenesis and raised GWP; high-C/N kiwifruit branches retarded decomposition, favoured Cmin accumulation, and lowered NGWP despite moderate C retention.
- Methane accounted for >99% of GWP in both treatments, overwhelming the N_2_O mitigation effect and making both RSD treatments net GHG sources after accounting for C-seq offsets.

**What are the implications of the main findings?**

- The C/N ratio, together with substrate decomposability, governs the trade-off between short-term SOC sequestration and GHG emissions: WS favours rapid C build-up at a higher warming cost, whereas KB achieves a smaller net climatic footprint.
- Selecting organic amendments for field RSD requires balancing carbon sequestration against methane emission costs; high-C/N materials such as kiwifruit branches may better align with low-carbon agricultural objectives.

**Abstract:**

**Background/Objectives**: Reductive soil disinfestation (RSD) is increasingly viewed as a viable alternative to chemical fumigation, yet the role of organic amendment quality in regulating greenhouse gas (GHG) fluxes, soil organic carbon (SOC) dynamics, and net climate forcing remains poorly understood. **Methodology**: In a 30-day anaerobic incubation experiment, we set up three treatments—a flooded control (CK), soil amended with wheat straw (WS, C/N = 55.5), and soil amended with kiwifruit branches (KB, C/N = 110.8)—each replicated three times under identical conditions. **Results**: Both WS and KB additions strongly stimulated CO_2_ and CH_4_ production, while suppressing N_2_O emissions by over 85% relative to CK. The WS treatment exhibited a substantially higher global warming potential (GWP, 823.52 t ha^−1^) than KB (636.23 t ha^−1^), with CH_4_ accounting for more than 99% of total GWP. Although WS surpassed KB in short-term carbon sequestration efficiency (25.79% vs. 20.11%) and showed greater hydrolytic enzyme activities (βG, CBH, and XYL), the two organic amendments diverged clearly in carbon fraction distribution: C_mic_ was 17.9% higher under WS, whereas C_min_ was 16.2% higher under KB. When factoring in the CO_2_ equivalent benefit derived from carbon sequestration, the net GWP (NGWP) indicated that both RSD treatments remained net GHG sources. Notably, KB yielded a markedly lower NGWP (611.60 t CO_2_-eq ha^−1^) than WS (798.19 t CO_2_-eq ha^−1^), highlighting a fundamental trade-off: WS favored rapid SOC accumulation at the expense of elevated methane emissions, whereas KB achieved a smaller climatic footprint despite more moderate carbon retention. **Conclusions**: These findings underscore that selecting organic amendments for field RSD requires balancing the competing goals of carbon sequestration and GHG mitigation.

## 1. Introduction

Intensive protected cultivation dominates vegetable production across China, which accounts for over 80% of global protected farmland. Its total planting area peaked at 4.0 million hm^2^ in 2010 and subsequently stabilised at 2.67 million hm^2^ by 2021 following structural agricultural adjustments [1,2,3]. Long-term continuous monocropping and excessive synthetic fertilisation trigger widespread soil salinisation, acidification, and microbial community degradation, which in turn aggravate soil-borne diseases and hinder sustainable crop production [4,5]. Conventional chemical soil fumigation can efficiently control pathogens but poses persistent environmental hazards, making reductive soil disinfestation (RSD) an eco-friendly alternative. Also known as anaerobic soil disinfestation (ASD) or biological soil disinfestation (BSD), RSD originated in the Netherlands and Japan [6,7]. This technique creates anaerobic conditions through organic amendment and flooding to suppress pathogens and improve soil properties. Within this framework, exogenous organic substrates act as the key factor regulating soil carbon transformation and greenhouse gas (GHG) fluxes [8,9].

Organic amendment quality, often indexed by C/N ratio but encompassing broader compositional attributes, modulates microbial metabolic pathways and generates a critical trade-off between GHG emissions and short-term soil organic carbon (SOC) retention—a central issue addressed in this study. Previous studies have shown that organic inputs stimulate microbial respiration and increase CO_2_ and CH_4_ production under the anaerobic conditions induced by RSD [10,11,12]. Meanwhile, excess nitrogen application generally accelerates N_2_O generation [8,13,14]. Plant residues with contrasting C/N values differ markedly in their decomposition rates, which further reshapes the partitioning of SOC between labile and physically protected pools [15,16]. Under anaerobic conditions, CH_4_ production is primarily driven by methanogenic archaea via acetoclastic and hydrogenotrophic pathways, while its oxidation is mediated by methanotrophs at oxic–anoxic interfaces [17,18]. The supply of labile carbon from organic amendments directly fuels these methanogenic communities, whereas the accompanying release of extracellular hydrolytic enzymes—such as β-1,4-glucosidase and cellobiohydrolase—governs the initial depolymerisation of polysaccharides into fermentable substrates, thereby coupling enzyme activity with subsequent GHG production [19,20]. However, few studies have systematically investigated how the C/N ratio of organic amendments regulates GHG trade-offs alongside short-term SOC sequestration under uniform RSD conditions.

Wheat straw and kiwifruit branches are representative agricultural wastes generated in the Guanzhong Plain. Wheat straw is produced in large quantities and is readily available, whereas a substantial amount of lignified kiwifruit pruning branches is generated annually but lacks effective utilisation pathways. To date, most RSD studies have focused on herbaceous crop residues, with limited attention given to woody orchard pruning materials, despite their contrasting C/N ratios and decomposition behaviours. By comparing these two locally representative substrates, this study provides a basis for the rational utilisation of regional agricultural wastes in RSD practices. However, whether the contrasting C/N ratios and lignocellulose compositions of herbaceous versus woody residues lead to fundamentally different trade-offs between short-term SOC retention and GHG emissions under anaerobic RSD conditions remains poorly understood. To address this knowledge gap, we conducted a 30-day anaerobic incubation under conditions designed to simulate field RSD, using low-C/N wheat straw (WS) and high-C/N kiwifruit branches (KB) as contrasting amendments. Two hypotheses were tested. (1) the labile carbon in WS should accelerate microbial mineralization, thereby increasing CO_2_ and CH_4_ emissions while promoting short-term SOC retention through rapid microbial immobilization. (2) the more recalcitrant KB should slow carbon turnover, dampen GHG release, and favour the build-up of physically protected SOC, albeit with lower sequestration efficiency.

Our study quantifies the changes in GHG fluxes and SOC fractions induced by these contrasting amendments, and examines the corresponding shifts in the activities of key hydrolytic enzymes (βG, CBH, XYL). The overall goal is to clarify how amendment C/N ratio shapes soil carbon transformation pathways and the associated GHG trade-offs, specifically from the perspectives of carbon fraction distribution and enzymatic function during anaerobic RSD.

## 2. Materials and Methods

### 2.1. Site Description and Soil Sampling

The experimental site is located at the protected cultivation base of Zhangbu Village, within the Yangling Agricultural High-tech Industrial Demonstration Zone in the central Guanzhong Plain, Shaanxi Province (108°3′41″ E, 34°15′51″ N). The region features a warm temperate semi-humid continental monsoon climate with an altitude of approximately 520 m, an annual average temperature of 13 °C, an annual average precipitation of 620 mm, and an annual average evaporation of approximately 1400 mm. The experimental soil was Lou soil, corresponding to Luvic Terric Anthrosols under WRB classification and Calcixerollic Xerochrepts (Inceptisols) in USDA Soil Taxonomy. This soil developed from loess parent material under long-term anthropogenic manure accumulation in the Guanzhong Plain of Shaanxi Province, China.

The solar greenhouse used in this study was constructed in 2012, covering an area of approximately 666.4 m^2^. It manages a single-cropping system, with tomato (*Solanum lycopersicum*) as the cultivated crop. The greenhouse has been continuously planted with tomatoes for over 10 years and suffers from severe soil-borne diseases, soil salinisation, reduced crop yields, and deteriorated vegetable quality.

Soil samples were collected from the topsoil layer (0–20 cm) during the summer fallow period on 24 June 2021. The fresh soil was naturally air-dried and cleared of visible impurities. A portion of the soil was reserved to determine basic soil physicochemical properties (Table 1); the remaining soil was ground and passed through a 2-mm sieve for the indoor incubation experiment.

The tested organic materials included WS collected from the Doukou Experimental Station in Sanyuan County, which was oven-dried and cut into segments shorter than 2 cm, and KB, derived from local kiwifruit orchard pruning waste that was oven-dried and pulverised after collection. The basic physicochemical properties of these materials are presented in Table 2.

### 2.2. Experimental Design

We carried out a single-factor completely randomized laboratory incubation trial with three distinct soil treatments: the flooding-only blank control (CK), soil mixed with wheat straw (WS), and soil incorporated with kiwifruit branches (KB). All plant residues were added at a rate of 2% relative to soil dry mass, and three independent replicate incubation bottles were set up for each treatment. We collected destructive soil samples on four separate dates throughout the 30-day incubation period to analyze variations in soil carbon and microbial-related indicators.

Before incubation, sieved soil samples (<2 mm) were weighed and placed into incubation bottles. The soils were pre-incubated at 25 °C in the dark for 3 days under moist conditions (70% water holding capacity) to restore microbial activity. After pre-incubation, the organic materials were added at a rate of 2% (*w*/*w* dry weight basis) and thoroughly mixed. Deionised water was then added to maintain a 4-cm flooding water layer above the soil surface. The 4-cm water layer added to the 500-mL gas sampling bottles corresponded to a liquid volume of approximately 270–290 mL, with slight variation between WS and KB due to differences in the bulk density of the added materials. Water holding capacity (WHC) was determined by the funnel method: about 20 g of soil was placed in a funnel lined with filter paper, saturated with distilled water, and left to drain for 2 h; the gravimetric water content after drainage was taken as WHC. Before flooding, soils were pre-incubated at 70% WHC for 3 days to restore microbial activity. Once the 4-cm water layer was added at the start of the 30-day incubation, the soils were fully submerged and remained saturated for the entire incubation period. The bottles were sealed, flushed with highly pure nitrogen gas, and incubated in the dark at 45 °C for 30 days. Following this incubation period, the overlying water was removed. After the soils were natural air-dried for 7 days, they were collected from the bottles for subsequent analysis.

Due to experimental requirements, incubation bottles of different specifications were used for gas sampling and destructive soil analysis. To ensure a consistent headspace volume during incubation, the ratio of soil dry weight to headspace volume was kept identical across all bottles. Specifically, soil equivalent to 300 g dry weight was placed into 1-L incubation bottles for periodic destructive sampling; at each sampling interval, these bottles were opened, the excess water was drained, and the soil was harvested. For gas sampling, soil equivalent to 150 g dry weight was filled into 500-mL anaerobic incubation bottles.

### 2.3. Sample Collection and Analytical Measurements

#### 2.3.1. Gas Sample Collection

Gas sampling for CO_2_, CH_4_, and N_2_O was conducted on days 1, 2, 3, 4, 5, 6, 7, 9, 11, 13, 15, 17, 19, 21, 23, 25, 28, and 30 of incubation. At each sampling interval, gas samples were extracted from the headspace of the anaerobic bottles using syringes and stored in gas-sampling bags. After each collection, each incubation bottle was opened to vent the internal gas and flushed with highly pure N_2_ to maintain strictly anaerobic conditions inside the bottles. Subsequently, the bottles were returned to the incubator for continuous anaerobic incubation.

#### 2.3.2. Soil Sample Collection

Destructive soil sampling was performed on days 0, 5, 14, 22, and 30 of incubation. During sampling, the soil in each bottle was thoroughly mixed, and undecomposed straw residues were removed. Half of the homogenised soil was air-dried, while the remaining fresh soil was refrigerated for subsequent analysis.

#### 2.3.3. Determination of Measurement Indices

Gas samples were collected using 20-mL plastic syringes fitted with three-way stopcocks, immediately transferred to laminated aluminium-plastic bags, and stored in the dark at room temperature. All samples were analysed within 9 h on an Agilent 7890A gas chromatograph (Agilent Technologies, Palo Alto, CA, USA) [21]. A 1-mL aliquot of headspace gas was manually injected through a six-port sampling valve fitted with a 1-mL sample loop. Gas separation was achieved on a HayeSep Q 80/100 column (2 m × 3 mm i.d.) in series with a molecular sieve 5A column (3 m × 2 mm i.d.). Concentrations of CO_2_, CH_4_, and N_2_O were determined by a flame-ionisation detector (FID) and an electron-capture detector (ECD). The FID was used for CH_4_ and CO_2_; CO_2_ was converted to CH_4_ by an in-line post-column methaniser prior to FID quantification, while N_2_O was measured by ECD. High-purity N_2_ served as carrier gas at 25 mL min^−1^. The column oven and FID were maintained at 55 °C and 300 °C, respectively. For N_2_O detection, make-up gas was 95% argon + 5% methane; the oven and ECD were set to 55 °C and 300 °C. A purge gas (10% CO_2_ balanced in N_2_) was supplied to the ECD to mitigate matrix interference. Detector calibration was performed against certified reference gas mixtures (National Research Centre for Standard Materials, China), with calibration ranges of 0.5–500 μL L^−1^ for CO_2_ and CH_4_, and 0.05–10 μL L^−1^ for N_2_O. Limits of detection (signal-to-noise ratio = 3:1) were 2.1 μL L^−1^ for CO_2_, 0.8 μL L^−1^ for CH_4_, and 0.02 μL L^−1^ for N_2_O. Relative standard deviations (*n* = 6) for repeated measurements of reference gases were 2.3% for CO_2_, 1.8% for CH_4_, and 3.5% for N_2_O.

Soil physicochemical properties were analysed in accordance with standard *Soil Agricultural and Chemical Analysis Methods* [22,23]. Total soil carbon (TC, g·kg^−1^) was analysed with a Vario EL cube elemental analyser (Elementar Analysensysteme GmbH, Langenselbold, Germany) via high-temperature dry combustion. Soil inorganic carbon (SIC, g·kg^−1^) was measured using the gas volumetric method, and SOC was determined using the external heating potassium dichromate oxidation method. Dissolved organic carbon (DOC) was extracted with ultrapure water. Microbial biomass carbon (MBC) was determined using the chloroform fumigation–K_2_SO_4_ extraction method.

Soil β-1,4-glucosidase (βG, nmol g^−1^ h^−1^), cellobiohydrolase (CBH, nmol g^−1^ h^−1^), and β-1,4-xylosidase (XYL, nmol g^−1^ h^−1^) activities were determined using the microplate fluorometric assay with 4-methylumbelliferyl (MUB)-linked substrates (βG: 4-methylumbelliferyl-β-D-glucopyranoside; CBH: 4-methylumbelliferyl-β-D-cellobioside; XYL: 4-methylumbelliferyl-β-D-xylopyranoside). Fluorescence intensity was measured with a microplate reader (Synergy H1, BioTek Instruments, Inc., Winooski, VT, USA) at an excitation wavelength of 365 nm and an emission wavelength of 450 nm. All assays were performed in triplicate, with substrate-free and enzyme-free controls included to correct for background fluorescence. Soil polyphenol oxidase (S-PPO, mg·g^−1^·d^−1^) was determined using commercial assay kits (catalogue no. RXWB0196-96; Ruixin Biotechnology Co., Ltd., Quanzhou, China).

Total soil nitrogen (TN) was digested with concentrated H_2_SO_4_ and then measured using a continuous flow analyser (Auto Analyser 3-AA3; SEAL Analytical, Mequon, WI, USA). Soil pH was measured with a SevenCompact S220 pH meter (Mettler Toledo, Greifensee, Switzerland) at a soil-to-water ratio of 1:2.5 (*w*/*v*). Soil electrical conductivity (EC) was determined using a SevenCompact S230 conductivity meter (Mettler Toledo, Greifensee, Switzerland) in a 1:5 (*w*/*v*) soil-to-water suspension. Ammonium nitrogen (NH_4_^+^-N) and nitrate nitrogen (NO_3_^−^-N) were extracted with a 2 M KCl solution and determined using the same continuous flow analyser system. The pH and EC values measured during incubation are provided in Supplementary Figure S1.

### 2.4. Data Analysis

#### 2.4.1. Calculation of GHG Emissions

The emission rates and cumulative emissions of the three GHGs (CO_2_, CH_4_, and N_2_O) were calculated according to the following formulas (Zheng et al., 2008) [21]:

(1) $$F=\rho \times \frac{∆C}{∆t}\times \frac{273.15}{\left(273.15+T\right)}\times \frac{V}{m}$$

(2) $$M=\sum (\frac{F_{i}+F_{i-1}}{2})\times (t_{i}-t_{i-1})\times 24,$$

where *F* represents the emission rate of CO_2_ (mg·kg^−1^·h^−1^), N_2_O (μg·kg^−1^·h^−1^), and CH_4_ (mg·kg^−1^·h^−1^); *ρ* is the gas density under standard conditions; Δ*C*/Δ*t* is the rate of concentration change in the headspace of the incubation bottle; *T* is the incubation temperature (°C); *V* is the headspace volume of the incubation bottle (m^3^); *m* is the dry weight of the incubated soil (kg); *M* is the cumulative emission; *t* is the sampling time (h); *t_i_* − *t_i_*_−1_ is the time interval between two adjacent samplings; and M(N_2_O), M(CH_4_), and M(CO_2_) are the cumulative emissions during the entire incubation period.

#### 2.4.2. Calculation of SOC Stock, Sequestration Efficiency, and Carbon Balance

The SOC stock (SOC_stock_), sequestered SOC (SOC_sequestered_, Mg ha^−1^), and CSE were calculated according to Srinivasarao et al. (2012) [24] using the following equations:

SOC_stock_ (Mg⋅ha^−1^) = SOC_content_ (g⋅kg^−1^) × soil depth (m) × bulk density (g⋅cm^−3^) × 10 (3)

SOC_sequestered_ (Mg ha^−1^) = Final SOC_stock_ (SOC_f_) − Initial SOC_stock_ (SOC_i_) (4)

C_SE_ (%) = SOC_sequestered_/Cumulative carbon input × 100% (5)

Additionally, the net C balance (NCB) and the actual carbon sequestration efficiency (ACSE) were calculated as follows:

NCB = (SOCT − SOCi) − (SOC0 − SOCi)(6)

ACSE (%) = Net C balance/Cumulative carbon input × 100(7)

where SOCi = initial soil organic carbon stock; SOCT = soil organic carbon stock of the treatments amended with organic materials; SOC0 = soil organic carbon stock of the non-amended control; and bulk density = 1.418 g cm^−3^.

#### 2.4.3. Operationally Defined Carbon Pools: CO_2_–CH_4_-Derived Labile C (C_mic_) and Microaggregate-Associated C (C_min_)

Based on the conceptual SOC fraction model [25,26,27], this study adopted a simplified grouping protocol to functionally separate soil organic carbon pools. Here, C_mic_ refers to potentially mineralisable labile carbon, which was quantified from the cumulative CO_2_ and CH_4_ emissions measured across the entire incubation period. This fraction is distinct from MBC, which was measured by chloroform fumigation. Conversely, C_min_ denotes organic carbon bound within fine soil aggregates smaller than 0.25 mm, representing organic fractions physically protected by microaggregates. Importantly, this grouping follows an operational definition specific to this incubation trial and should not be equated with mineral-associated organic carbon (MAOM) or in-situ bioavailable SOC derived from conventional physical fractionation approaches. Throughout this paper, C_mic_ and C_min_ are used as operationally defined pools specific to this incubation trial, and should not be interpreted as equivalent to conventionally fractionated carbon pools (e.g., MBC or MAOM).

#### 2.4.4. Calculation of Soil Enzyme Activities

Soil hydrolase activities (nmol⋅g^−1^⋅h^−1^) were calculated based on the fluorescence values obtained from the microplate fluorometric assay using the following equations:

(8) $$\frac{F\times V}{e\times V_{1}\times t\times m}\;$$

(9) $$F=\frac{f-f_{b}}{q-f_{s}}\;$$

(10) $$e=\frac{f_{r}}{c_{s}\times V_{2}}\;$$

(11) $$q=\frac{f_{q}-f_{b}}{f_{r}}$$

where *F* is the corrected fluorescence value of the sample; *V* is the total volume of soil suspension (mL); *V*_1_ is the volume of soil suspension added to each microplate well (mL); *t* is the incubation time (h); *m* is the dry mass of the soil (g, converted from fresh soil weight); *f* is the fluorescence value of the sample wells; *f_b_* is the fluorescence value of the blank wells; *q* is the quenching coefficient; *f_s_* is the fluorescence value of the negative control; *e* is the fluorescence emission coefficient; *f_r_* is the fluorescence value of the reference standard wells; *C_s_* is the concentration of the reference standard (nmol⋅mL^−1^); *V*_2_ is the volume of the added reference standard solution (mL); *f_q_* is the fluorescence value of the quenching standard wells.

#### 2.4.5. Global Warming Potential (GWP) and Net Global Warming Potential (NGWP)

According to the Intergovernmental Panel on Climate Change (IPCC) 100-year GWP characterisation factors, the GWP values of methane (CH_4_) and nitrous oxide (N_2_O) are 28 and 265 times greater than that of carbon dioxide (CO_2_) [8]. Accordingly, the total GWP was calculated as follows:

GWP = W_CO_2__ + 28 W_CH_4__ + 265 W_N_2_O_ (12)

where W_CO_2__, W_CH_4__, and W_N_2_O_ represent the total cumulative emissions of CO_2_, CH_4_, and N_2_O throughout the incubation period.

To evaluate the climate mitigation potential of organic matter retention, the CO_2_ equivalent of soil carbon sequestration (C_seq-CO2-eq) was calculated using the following equation:

C_seq-CO_2_-eq = [C seq (g pot^−1^)/soil mass (g pot^−1^)] × 3.67 × (BD × D × 10) (13)

where soil mass = 300 g pot^−1^, BD = bulk density (1.418 g cm^−3^), D = depth (20 cm), and soil mass in each pot was 300 g.

Finally, the NGWP was estimated by combining total GWP and the carbon sequestration offset:

NGWP = GWP_total − C_seq-CO_2_-eq (14)

The C-seq term represents the CO_2_ equivalent of the net SOC increase over the 30-day incubation, and is included here to assess the potential offset of GHG emissions within this timeframe. Under the short-term (30 d) and high-temperature (45 °C) conditions of this laboratory trial, the rapid mineralisation of labile organic carbon into CH4 can heavily influence the net climate outcome, which remains highly dependent on how the added carbon is partitioned between immediate gas emissions and stable SOC formation. The NGWP results are thus best interpreted as time-bound estimates within the experimental timeframe.

### 2.5. Statistical Analysis

The experimental data were compiled using WPS Office (Kingsoft Office Software, Singapore). One-way analysis of variance (ANOVA) was performed using SPSS 22.0 to analyse GHG emissions, soil carbon and nitrogen fractions, and other physicochemical parameters, while soil enzyme activities were evaluated using two-way ANOVA. Multiple comparisons of means were conducted using the least significant difference (LSD) test at a significance level of *p* < 0.05. Omnic 8.0 (Thermo Fisher Scientific, Waltham, MA, USA) and Origin 2021 (OriginLab, Northampton, MA, USA) were used to perform correlation analyses among SOC, nitrogen components, GHG fluxes, enzyme activities, and other parameters. All figures and tables presented in this study were generated using Origin 2021.

## 3. Results

### 3.1. GHG Emission Rates and Cumulative Emissions

**Figure 1:**
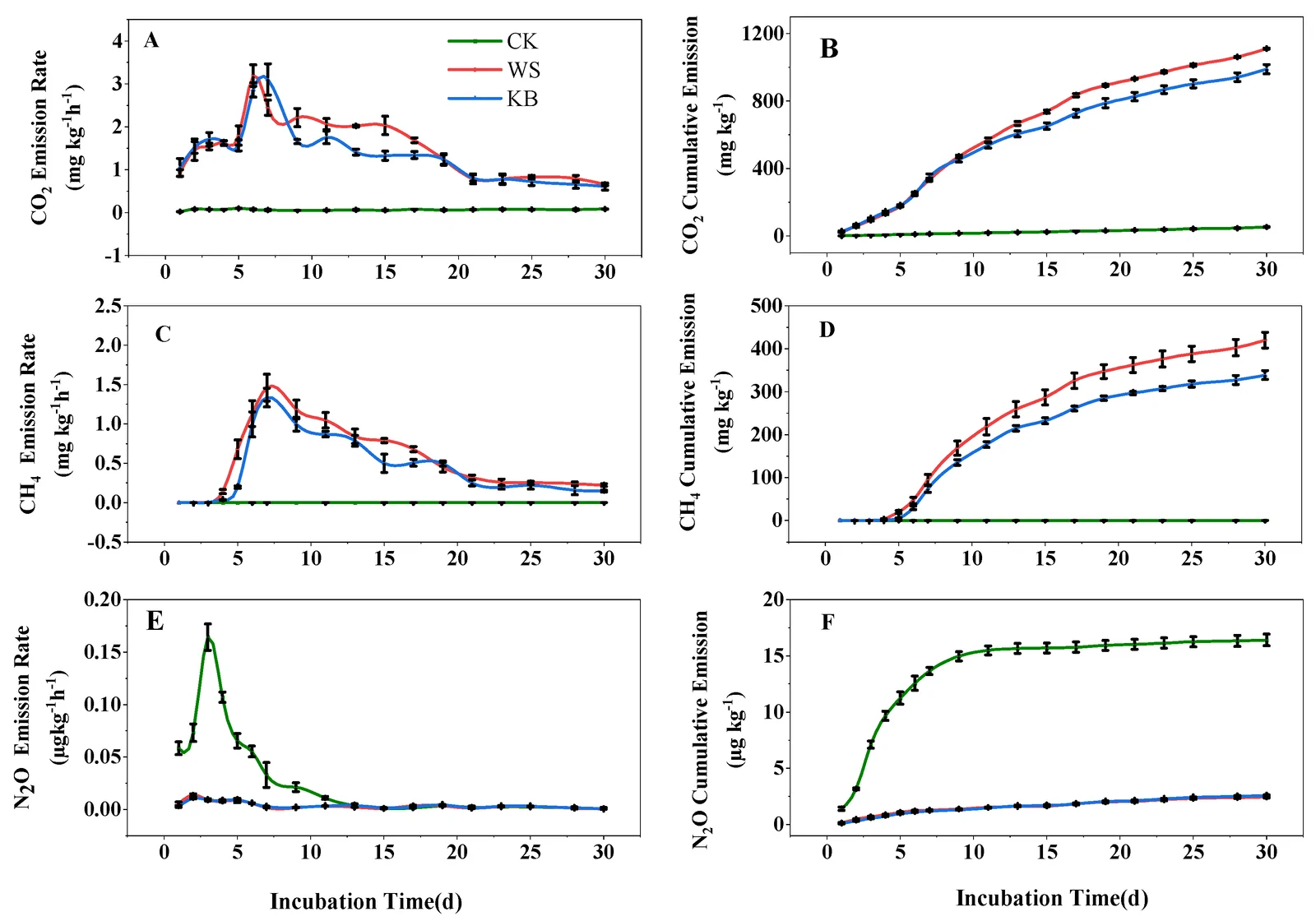
Effects of different organic materials on greenhouse gas emission rates and cumulative emissions. Emission rate and cumulative emission of soil CO_2_ (**A**,**B**), CH_4_ (**C**,**D**), and N_2_O (**E**,**F**).

Figure 1 illustrates the temporal dynamics of CO_2_, CH_4_, and N_2_O emission rates and cumulative fluxes under the CK, WS, and KB treatments during the 30 days of flooded anaerobic incubation. CO_2_ emission rates (Figure 1A) initially increased before gradually declining across both organic amendment groups. Emissions in the WS and KB treatments rose rapidly after 3 days, peaking on day 6 for WS (3.20 mg·kg^−1^·h^−1^) and day 7 for KB (3.11 mg·kg^−1^·h^−1^). From day 3 to day 30, both WS and KB exhibited significantly higher CO_2_ emission rates than CK, with WS consistently outperforming KB. Cumulative CO_2_ emissions (Figure 1B) in WS (1111.81 mg·kg^−1^) were significantly higher than those in KB (989.55 mg·kg^−1^, *p* < 0.05), representing 21.0- and 18.7-fold increases relative to CK (53.02 mg·kg^−1^), respectively. This indicates stronger carbon mineralisation promotion by the low-C/N WS.

CH_4_ dynamics (Figure 1C,D) broadly mirrored those of CO_2_ but exhibited a temporal delay. Emissions in the WS and KB treatments were negligible during the first 4 days, increased sharply after day 5, peaked on day 7 (1.46 and 1.34 mg·kg^−1^·h^−1^, respectively), and then stabilised after day 20. Cumulative CH_4_ production in WS (424.47 mg·kg^−1^) was 24% higher than in KB (322.78 mg·kg^−1^), with both treatments far exceeding CK. This demonstrates that plant-derived carbon strongly stimulated methanogenesis with clear, material-dependent effects.

In contrast, N_2_O emissions (Figure 1E,F) showed the opposite pattern. Peaks occurred on days 2 and 3, with CK (0.16 μg·kg^−1^·h^−1^) exhibiting substantially higher emissions than WS (0.014 μg·kg^−1^·h^−1^) or KB (0.011 μg·kg^−1^·h^−1^). Emissions subsequently stabilised after day 12, driven by initial nitrate-fuelled denitrification followed by substrate depletion. Cumulative N_2_O emissions in CK (16.40 μg·kg^−1^) were 6.72- and 6.41-fold higher than those in the WS and KB treatments, respectively, indicating profound N_2_O suppression via microbial nitrogen mining in the amended soils. Temporal differences between WS and KB were largely attributed to their contrasting C/N ratios; the lower C/N WS decomposed faster during the early phase, whereas the high-C/N KB underwent gradual mineralisation during the later stage.

### 3.2. Soil Carbon Fractions

**Figure 2:**
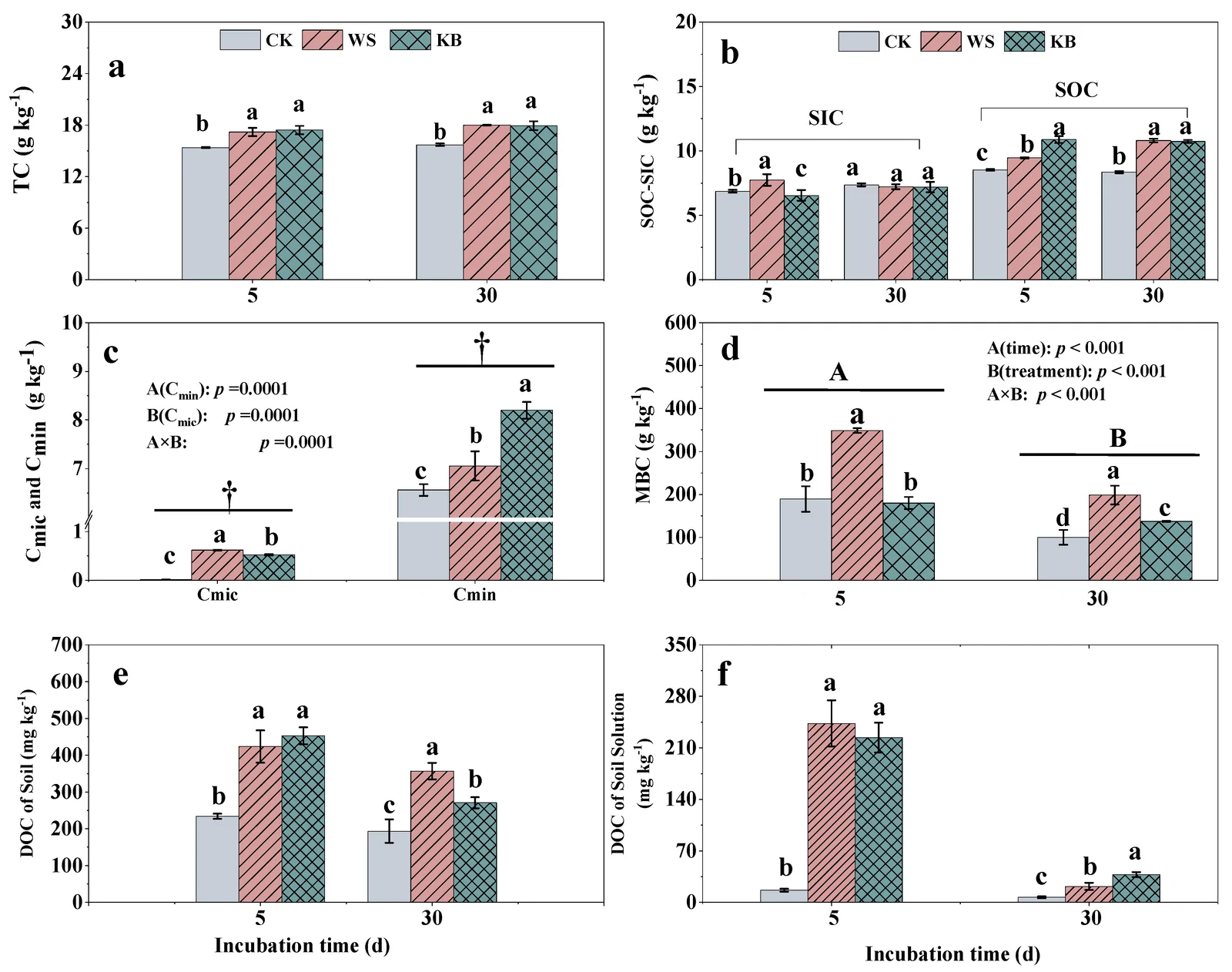
Effects of different organic amendments on stable and labile soil carbon fractions. (**a**) Total soil carbon (TC); (**b**) soil organic carbon (SOC) and soil inorganic carbon (SIC); (**c**) mineralizable labile C derived from cumulative GHG emissions (C_mic_) and aggregate-protected organic C in microaggregates (<0.25 mm) (C_min_); (**d**) soil microbial biomass carbon (MBC); (**e**,**f**) dissolved organic carbon (DOC) in the soil and incubation solution, respectively. Explanation of significance labels: lowercase letters = inter-treatment difference at fixed incubation time; uppercase letters = intra-treatment difference between 5 d and 30 d; † = significant differences between the two carbon fractions within the same subpanel (*p* < 0.05).

Figure 2 systematically illustrates the effects of different treatments on soil carbon fractions, MBC, and DOC following 30 days of anaerobic incubation experiment. Exogenous plant-derived organic carbon (WS and KB) significantly increased TC at both 5 and 30 days, with no significant difference observed between WS and KB (Figure 2a). This indicates that under short-term flooding, exogenous carbon input effectively enhanced the soil carbon pool, various carbon sources exerted similar carbon-accumulation effects, along with limited carbon loss without exogenous carbon addition.

Figure 2b shows that at the early stage (5 days of incubation), WS increased SIC and SOC by 12.74% and 11.02% compared with the CK, respectively; KB decreased SIC by 4.70% and increased SOC by 27.80%. At the late stage (30 days), no significant difference in SIC was observed among treatments. This is likely because the calcareous soil used in the experiment is rich in calcium ions, which reacted with CO_2_ released from organic matter decomposition during the early stage to form calcium carbonate, resulting in elevated SIC that stabilised thereafter. SOC showed a similar trend to that of TC, with carbon-amended treatments exhibiting significantly higher SOC than the CK (*p* < 0.05), and no significant difference between WS and KB.

C_min_ and C_mic_ in WS and KB were both significantly higher than those in the CK after 30 days (*p* < 0.05; Figure 2c). C_mic_ in WS was 17.9% higher than that in KB, while C_min_ in KB was 16.2% higher than that in WS. Relative to the CK, C_min_ in WS and KB increased by 7.53 and 24.98%, respectively, while C_mic_ increased 43- and 36-fold. This confirms that exogenous carbon simultaneously improves both labile and stable carbon pools. In addition, C_min_ was significantly higher than C_mic_ in all treatments. The C_min_/C_mic_ ratio was 467 in the CK, but only 12 and 16 in WS and KB, respectively, indicating that exogenous carbon input markedly increases the proportion of the labile carbon pool and accelerates carbon turnover.

MBC was significantly higher at the early stage (5 days of incubation) than at the late stage (30 days) (*p* < 0.05; Figure 2d), reflecting greater carbon utilisation by anaerobic microorganisms during the early stage, followed by reduced microbial activity as labile organic carbon became depleted. MBC in WS was significantly higher than that in the CK and KB at both stages (*p* < 0.05). At the early stage, MBC in WS was 181.93 and 159.54 mg·kg^−1^ higher than that in CK and KB, respectively. At the late stage, WS and KB increased MBC by 98.44 and 74.4 mg·kg^−1^, respectively, compared with the CK, with the values following the trend of WS > KB. This pattern was consistent with C_mic_, indicating that WS is more readily utilised by soil microorganisms.

Figure 2e,f show the DOC dynamics. No significant difference in soil DOC was observed between WS and KB at the early stage, but soil DOC in WS was significantly higher than that in KB at 30 days. In contrast, solution DOC was higher in WS at 5 days but higher in KB at 30 days (*p* < 0.05). This difference was attributed to the distinct decomposition characteristics of the two carbon sources: labile components in WS were released rapidly during the early stage, resulting in higher solution DOC in WS. In the later stage, labile fractions in WS were exhausted, while KB decomposed gradually, leading to divergent patterns of soil and solution DOC.

### 3.3. SOC Sequestration

**Figure 3:**
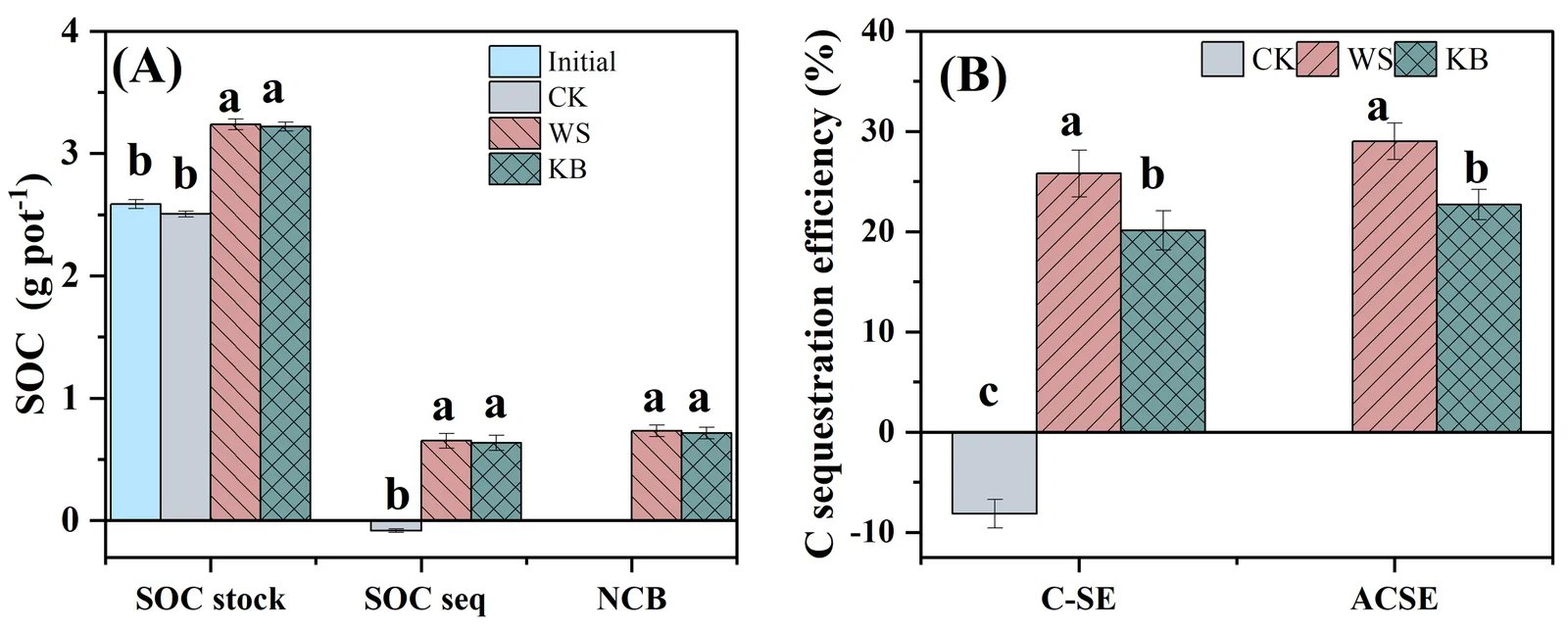
Effect of different organic materials on soil organic carbon stocks and organic carbon sequestration rate. (**A**) Carbon stocks. (**B**) Carbon immobilisation rate. Lowercase letters represent significant differences between treatments.

Figure 3 shows changes in SOC stock and carbon sequestration after 30 days of anaerobic incubation under RSD. As shown in Figure 3A, SOC in the CK treatment fell from the initial 2.587 g pot^−1^ to 2.505 g pot^−1^, with a carbon sequestration value of −0.08 g pot^−1^. This outcome suggests that anaerobic flooding accelerated the mineralisation of native SOC when no organic material is added. By contrast, WS and KB additions raised SOC stocks to 3.238 g pot^−1^ and 3.220 g pot^−1^, representing increases of 25.18% and 24.49%, respectively. Their measured carbon sequestration reached 0.65 g pot^−1^ and 0.63 g pot^−1^; with no significant difference observed between WS and KB. The net carbon balance (NCB) was 0.73 g pot^−1^ for WS and 0.71 g pot^−1^ for KB, demonstrating that organic inputs can offset native soil carbon losses.

As shown in Figure 3B, the SOC sequestration efficiency (C_SE_) and actual carbon sequestration efficiency (ACSE) shared identical variation patterns, following the order WS > KB > CK (*p* < 0.05). The CK had a negative sequestration rate of −8.20%, owing to continuous net SOC mineralisation. WS had a significantly higher C-SE (25.79%) and ACSE (29.01%) than KB (20.10% and 22.70%). The low-C/N WS decomposes rapidly to fuel microbial assimilation and short-term carbon fixation, whereas the high-C/N KB break down slowly and favour stable SOC accumulation. Overall, the C/N ratio of applied organic materials largely determines SOC pool variation and sequestration capacity under RSD.

The addition of organic materials significantly altered the warming potentials (GWP) of individual GHGs and the overall climatic effect (Table 3).

Compared with the CK, organic amendments markedly increased the GWP of CO_2_ and CH_4_ while substantially reducing that of N_2_O. The CO_2_-related GWP was 2.75 t CO_2_-eq ha^−1^ in the WS treatment, which was significantly higher than the 2.37 t CO_2_-eq ha^−1^ observed in the KB treatment. The CH_4_-related GWP reached 820.72 t CO_2_-eq ha^−1^ under WS, which was 29.5% higher than the 633.82 t CO_2_-eq ha^−1^ in KB. In both amended treatments, CH_4_ was the dominant contributor to total GWP, accounting for more than 99% of the total value. By contrast, the N_2_O-related GWP dropped to approximately 0.04 t CO_2_-eq ha^−1^ in both WS and KB, representing an increase over 85% in mitigation compared with the CK (0.28 t CO_2_-eq ha^−1^).

In terms of total GWP, the WS treatment recorded 823.52 t CO_2_-eq ha^−1^, which was significantly higher than the 636.23 t CO_2_-eq ha^−1^ in KB, while the CK only reached 0.45 t CO_2_-eq ha^−1^. After correcting for the CO_2_ equivalent derived from net soil carbon sequestration (C-seq), the NGWP was calculated. The CK treatment showed a negative C-seq due to net mineralisation of native SOC, leading to an NGWP of 3.23 t CO_2_-eq ha^−1^. The C-seq values were 25.33 and 24.63 t CO_2_-eq ha^−1^ for WS and KB, respectively, resulting in corrected NGWP values of 798.19 and 611.60 t CO_2_-eq ha^−1^. Although both organic amendments remained net ghg sources, the KB treatment exhibited a notably lower net climatic footprint than WS.

**Figure 4:**
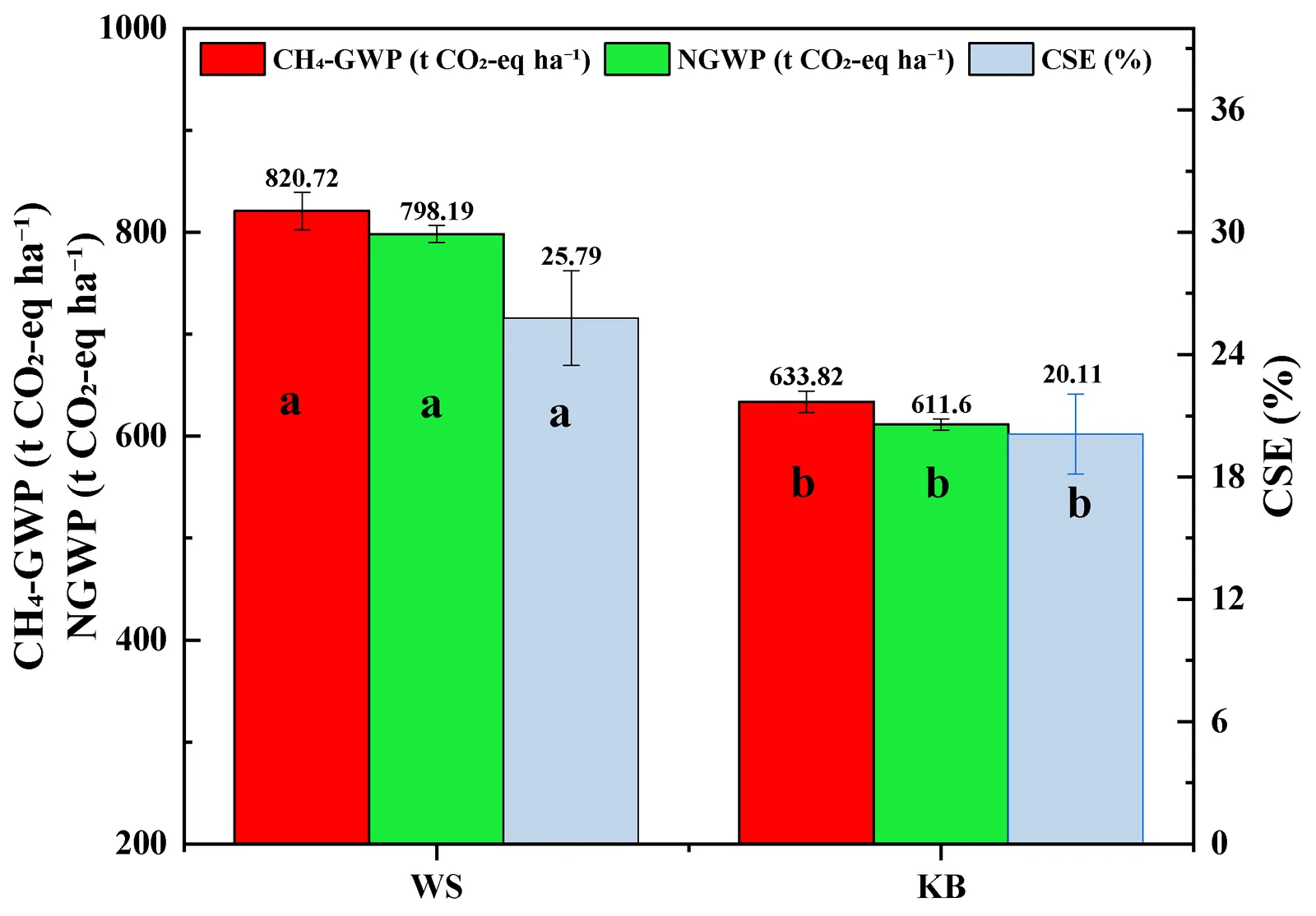
Trade-off between carbon sequestration efficiency and methane-induced warming potential under different organic amendments. Notes: Bars represent mean values (*n* = 3). CSE is shown on the right axis; CH_4_-GWP and NGWP are shown on the left axis. Lowercase letters indicate significant differences (*p* < 0.05) among treatments within the same variable (same color). Error bars indicate standard deviations. WS, wheat straw; KB, kiwifruit branches; CSE, carbon sequestration efficiency; CH_4_-GWP, methane global warming potential; NGWP, net global warming potential.

To better visualize the trade-off between carbon sequestration gain and methane emission cost, we plotted CSE alongside CH_4_-GWP and NGWP in a combined figure (Figure 4). WS showed a higher CSE (25.79%) than KB (20.10%), yet its CH_4_-GWP reached 820.72 t CO_2_-eq ha^−1^, far exceeding that of KB (633.82 t CO_2_-eq ha^−1^). After deducting the CO_2_ equivalent of carbon sequestration, WS still exhibited an NGWP of 798.19 t CO_2_-eq ha^−1^, roughly 30.5% higher than KB (611.60 t CO_2_-eq ha^−1^). These results indicate that the low-C/N material (WS) promoted short-term carbon sequestration at the cost of substantially elevated methane emissions, whereas the high-C/N material (KB) achieved a smaller net climatic footprint despite its more modest carbon retention.

### 3.4. Soil Enzyme Activities

**Figure 5:**
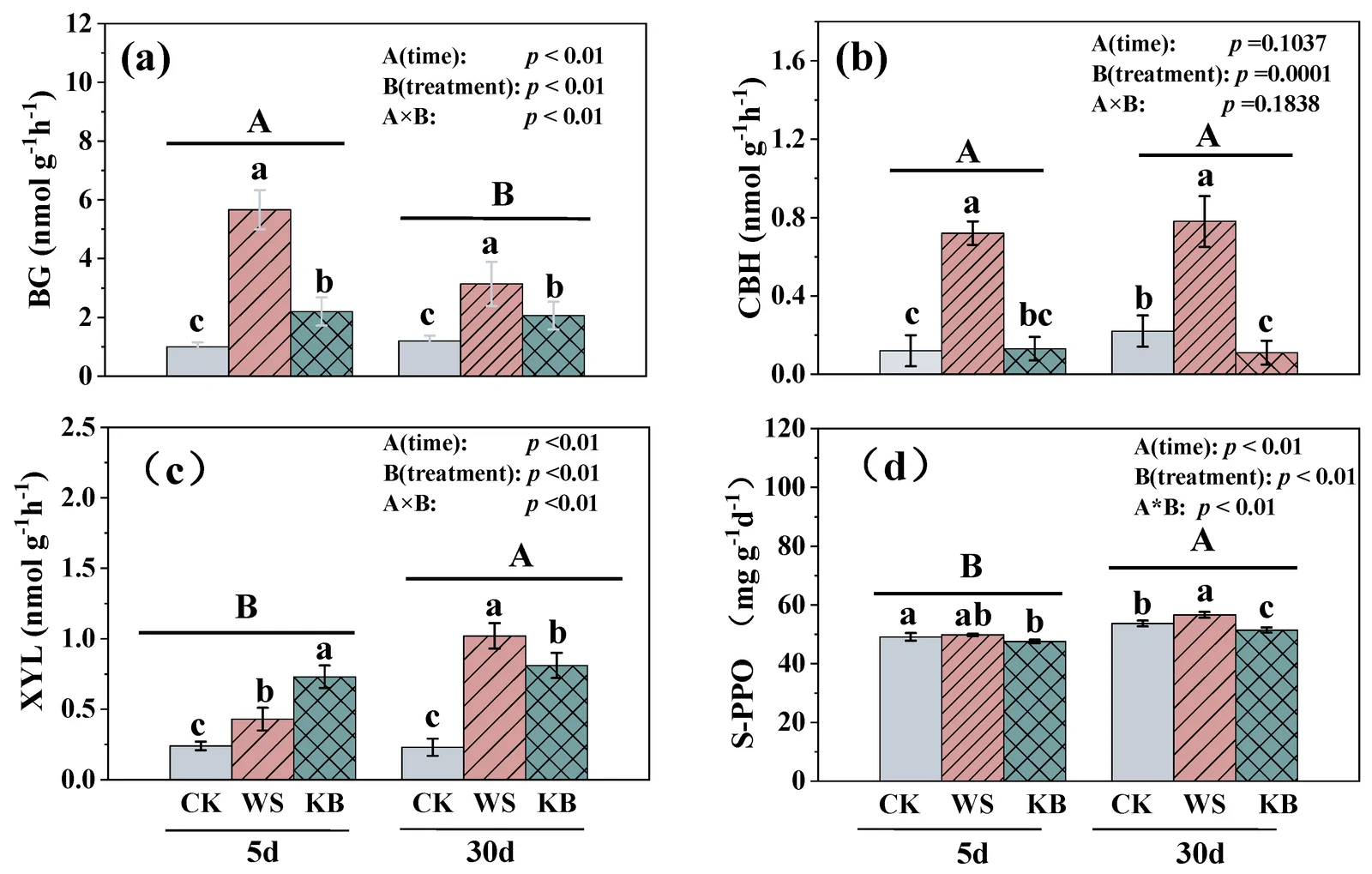
Effects of different organic amendments on soil extracellular enzyme activities. (**a**) β-1,4-glucosidase; (**b**) β-D-cellobiosidase; (**c**) β-1,4-xylosidase; (**d**) polyphenol oxidase. Notes: Lowercase letters = inter-treatment differences at fixed incubation time; uppercase letters = intra-treatment differences between incubation durations of 5 d and 30 d (*p* < 0.05, *n* = 6); The grey bars in the figure represent the CK treatment, the red bars represent WS, and the green bars represent KB.

The dynamic effects of different organic material amendments on the activities of key enzymes involved in soil carbon cycling under flooded anaerobic conditions are shown in Figure 5. A two-way ANOVA revealed that incubation time, treatment, and their interaction had highly significant effects on the activities of most enzymes (*p* < 0.01). Specifically, βG activity decreased significantly with prolonged incubation, and the addition of exogenous carbon significantly increased βG activity. The promotional effect of WS was significantly superior to that of KB (*p* < 0.05); βG activity in WS was 157 and 52% higher than that in KB on days 5 and 30, respectively. In contrast, CBH activity showed no significant temporal difference, but the treatment effect was consistent with that of βG, with the activity in WS being approximately 4.5-fold higher than that in KB, both of which were significantly higher than that in the CK.

XYL activity increased significantly with incubation time and exhibited a pronounced time–treatment interaction effect. The XYL activity in KB was 69.8% higher than in WS at the early incubation stage (day 5), whereas it was 26% higher in WS than that in KB at the late stage (day 30), which is consistent with the dynamic change pattern of DOC. S-PPO activity increased significantly with incubation time; however, activity in all treatments decreased significantly compared with the initial value at the early stage. Notably, activity in WS was significantly higher than that in the CK and KB at the late stage, indicating that WS addition could significantly increase S-PPO activity during the later period. Taken together, exogenous organic material amendments generally and significantly increased the activities of soil carbon-degrading enzymes by providing sufficient substrates for enzymatic reactions. Due to differences in their physicochemical properties, different carbon sources exhibited significant temporal differentiation in their regulatory effects on the activities of different types of enzymes. Among them, WS was more likely to promote the activities of hydrolases related to labile carbon decomposition, while KB had a more prominent promotional effect on XYL activity at the early stage, providing direct evidence to elucidate the enzymatic mechanism of organic carbon transformation under flooded conditions.

### 3.5. Correlations Among Soil Carbon Fractions, Carbon-Cycling Enzymes, and Soil Properties

**Figure 6:**
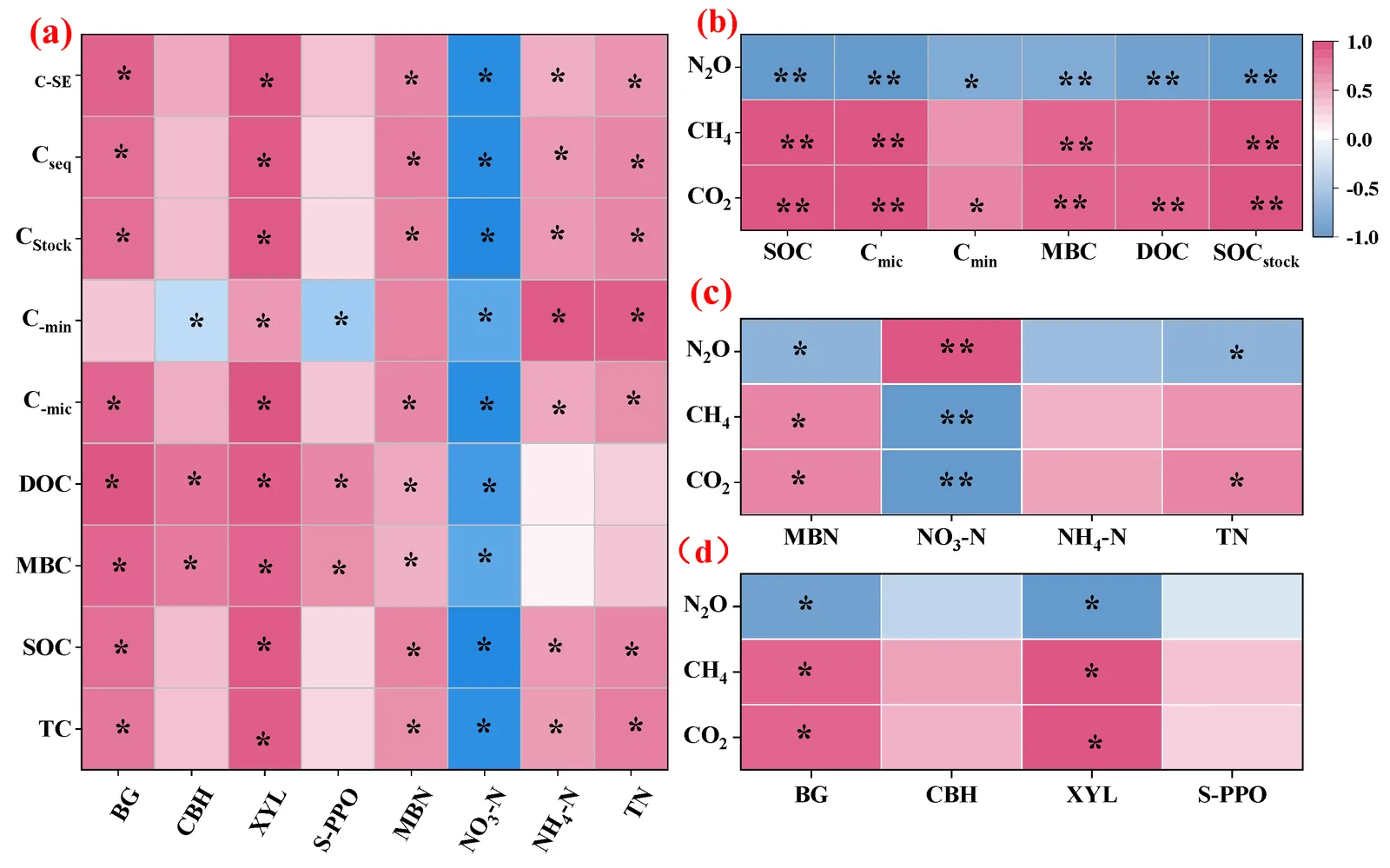
Correlation between soil carbon fractions, key carbon-cycling enzymes, greenhouse gas emissions, and soil nitrogen properties under flooded anaerobic conditions. * *p* < 0.05, ** *p* < 0.01. (**a**) Correlations among carbon fractions, nitrogen fractions and enzyme activities; (**b**) Correlations between greenhouse gases and carbon fractions; (**c**) Correlations between greenhouse gases and nitrogen fractions; (**d**) Correlations between greenhouse gases and enzyme activities.

The intrinsic linkages among soil carbon fractions, key carbon-cycling enzymes, GHG emissions, and soil nitrogen properties under flooded anaerobic conditions are shown in Figure 6. Except for C_min_, all other carbon fractions [TC, SOC, MBC, DOC, C_mic_, carbon stock (C_Stock_), sequestered carbon (C_seq_), carbon sequestration efficiency (C_SE_)] were significantly positively correlated with the activities of βG and XYL. Meanwhile, MBC and DOC also showed significant positive correlations with the activities of CBH and S-PPO, indicating that labile carbon fractions are the core substrates driving the increase in soil carbon-degrading enzyme activities. Soil carbon fractions were significantly negatively correlated with NO_3_^−^-N, but significantly positively correlated with TN, NH_4_^+^-N, and microbial biomass nitrogen (MBN), reflecting that carbon input promotes microbially mediated carbon–nitrogen coupling processes by regulating nitrogen transformation.

Figure 6b,c present the correlation between GHG emissions and soil carbon and nitrogen fractions. CO_2_ and CH_4_ emissions were significantly positively correlated with all soil carbon fractions, whereas N_2_O emissions were significantly negatively correlated with soil carbon fractions. CO_2_ and CH_4_ were significantly positively correlated with MBN, whereas N_2_O was significantly negatively correlated with MBN. Additionally, CO_2_ and CH_4_ were significantly negatively correlated with NO_3_^−^-N, whereas N_2_O was significantly positively correlated with NO_3_^−^-N; furthermore, the three GHGs showed weak correlations with NH_4_^+^-N and TN. These results indicate that exogenous carbon input, by enhancing the labile carbon pool and promoting microbial nitrogen immobilisation, intensifies CO_2_ and CH_4_ emissions while significantly inhibiting N_2_O release driven by denitrification.

Figure 6d further clarifies the correlations between GHG emissions and carbon-cycling enzymes. CO_2_ and CH_4_ emission fluxes were significantly positively correlated with the activities of βG and XYL, and were significantly negatively correlated with N_2_O emissions; in contrast, none of the three GHGs showed significant correlations with CBH or S-PPO activities. These correlations suggest that hydrolases associated with labile carbon decomposition are closely linked to soil carbon mineralisation and GHG emissions under flooded conditions. The strength of these associations is consistent with an enzymatic driving role in organic carbon transformation, although direct causal verification would require targeted experiments beyond the correlative approach used here.

## 4. Discussion

### 4.1. Linking Organic C/N Ratio to GHG Trade-Offs Through Enzyme Activities and Carbon Fractions Under RSD

Adding different organic materials to soil under RSD produced markedly different GHG emission patterns. In CK, cumulative CO_2_ stayed low throughout. WS raised cumulative CO_2_ about 21-fold over CK, while KB gave a nearly 19-fold increase. CH_4_ emissions ran roughly 24% higher with WS than with KB. Both amendments cut N_2_O release by >85% versus CK, and cumulative N_2_O from CK exceeded that from either amended treatment by more than six times.

The 100-year GWP for WS came to 823.52 t CO_2_-eq ha^−1^, roughly 30% above KB (636.23 t CO_2_-eq ha^−1^). CH_4_ dominated total GWP in both treatments (>99%), with CO_2_ and N_2_O contributing little (Table 3). These patterns reflect how WS and KB differentially altered the enzyme systems that drive C turnover. WS, with its low C/N and abundant soluble sugars and hemicellulose, exhibited markedly higher βG and CBH activities. This enzymatic response coincided with elevated CH_4_ emissions, a pattern consistent with the established view that enhanced hydrolysis increases the supply of fermentable substrates for methanogenic archaea [28,29,30]. Alongside this, WS produced a 17.9% larger C_mic_ pool, suggesting that much of the mineralized C remained in a microbially accessible but labile form. KB followed a different path: its higher lignin content and C/N ratio steered decomposition toward xylan breakdown, as reflected in the 69.8% higher XYL activity at day 5. This slower, more recalcitrant-oriented route limited the supply of methanogenic substrates, cutting CH_4_ emissions by 24%, while promoting physically protected carbon (C_min_ rose by 16.2% in KB). In essence, the C/N ratio set up a clear trade-off: WS pushed C through a labile, gas-prone pathway—boosting both CH_4_ emission and short-term C gain—whereas KB directed more C into stable pools, reducing the net warming impact.

Most earlier flooding studies were run at ambient temperature. Our higher incubation temperature (45 °C) accelerated C turnover, boosting methane’s share of total GWP [12,13]. All amended treatments substantially cut N_2_O emissions, an effect we attribute to microbial N mining under anaerobic conditions. The soil had low initial N and received no external N fertilizer during incubation. Carbon addition raised MBC and, with it, microbial N demand, so microbes took up nitrate and left less substrate for denitrification. At the same time, strongly reducing conditions stimulated nosZ-type denitrifiers, which reduce N_2_O completely to N_2_ [31,32]. The C/N ratio of the added material made little difference to the extent of N_2_O reduction—all RSD treatments showed comparable decreases. Carbon addition seems to trigger N mining regardless of substrate C/N; the C/N ratio matters more for the rate and route of C decomposition than for N consumption or N_2_O production.

According to the IPCC (2006) [33], the 100-year GWP of CH_4_ and N_2_O are 28 and 265 times that of CO_2_. Even though absolute CH_4_ emissions are lower than CO_2_, methane’s high GWP means that a modest rise in CH_4_ can cancel out the climate benefit from cutting N_2_O. This highlights a key drawback of RSD: while it enhances C sequestration and suppresses N_2_O, it also drives substantial CH_4_ release. Whether this trade-off can be resolved through operational adjustments remains an open question [8,33]. The 45 °C incubation used here does reflect summer RSD field temperatures, but such high heat favours thermophilic methanogens (e.g., Methanothermobacter) and speeds up methanogenesis beyond what would occur under mesophilic field conditions [34]. The magnitude of CH_4_ emissions recorded here—424.47 mg kg^−1^ for WS and 322.78 mg kg^−1^ for KB—exceeds the ranges commonly reported from RSD field trials. In field settings, CH_4_ typically contributes only a minor share to total GWP [8,11], whereas in our closed incubation system, CH_4_ dominated the warming potential. This disparity is likely attributable to three interacting factors: the elevated incubation temperature (45 °C vs. typical summer field temperatures of 25–35 °C), which promotes thermophilic methanogens; the sealed anaerobic bottle system, which restricts CH_4_ oxidation that would otherwise occur at oxic–anoxic interfaces in field soils; and the organic amendment rate (2%, *w*/*w*), which provided abundant labile carbon as substrate for methanogenesis. Together, these conditions suggest that our GWP estimates represent upper-end values rather than direct proxies for field performance. Regarding the enzyme–gas relationships, we recognise that the observed correlations alone do not demonstrate causation. Nonetheless, the strong associations between βG/XYL activities and CH_4_ emissions are consistent with a well-documented pathway in which hydrolytic enzymes depolymerise polysaccharides into low-molecular-weight substrates, which are then fermented to organic acids and ultimately converted to CH_4_ by methanogens [28]. Our data thus offer supportive—though not conclusive—evidence for this pathway, and we have framed our conclusions accordingly. For this reason, the GWP values reported here should be viewed as high-end estimates, and caution is needed when extending them to ambient-temperature scenarios.

### 4.2. Organic C/N Ratio Shapes Carbon Fractions and Sequestration Pathways Under RSD

The C/N-driven divergence in enzyme activities and carbon turnover pathways described above also fundamentally redirected the partitioning of newly incorporated carbon between labile and physically protected pools. After the RSD incubation experiment, the soil carbon fraction data showed clear differences among treatments. Specifically, the potentially mineralizable carbon (C_mic_) content in the wheat straw (WS) treatment was 17.9% higher than in the kiwifruit branch (KB) treatment. In contrast, for fine-aggregate-associated carbon (C_min_), however, the KB treatment exceeded the WS treatment by 16.2%. In terms of C_SE_, the WS treatment reached 25.79%, which was notably higher than the 20.11% recorded for KB. Because the CK received no external carbon input, its native SOC underwent net mineralisation, yielding a negative CSE of −8.20% (see Figure 3 and Figure 4). Our soil enzyme activity results confirmed these results. Throughout the entire incubation period, the activities of hydrolytic enzymes involved in labile carbon decomposition—such as *β*G and CBH—remained consistently higher in the WS treatment. In the early stage of the experiment, however, xylosidase (XYL) activity was actually higher in the KB treatment (Figure 5). Interestingly, this dynamic change in enzyme activity directly reflected the differences between the two organic materials in carbon decomposition and carbon pool distribution.

We can further analyse these findings from the perspectives of carbon priming effects and carbon stabilization mechanisms. The C/N ratio of wheat straw is moderately low, fitting well with the nutrient requirements for microbial growth. This promoted rapid microbial proliferation and also stimulated the synthesis and release of hydrolytic enzymes. The added carbon was quickly mineralized and turned into microbial residues, which became the main source of C_mic_. At the same time, this process induced a positive priming effect on native SOC. Overall, because the rate of microbial carbon fixation exceeded the rate of native carbon mineralization, this treatment achieved a higher carbon sequestration efficiency over the short term [35,36]. In contrast, kiwifruit branches have a higher C/N ratio and are rich in lignin. Nitrogen limitation suppressed microbial metabolism, slowing down the overall decomposition of organic matter [37,38]. A large amount of recalcitrant material became associated with soil clay particles and microaggregates, forming physically protected Cmin, and the mineral-associated carbon stock gradually increased. However, the slow decomposition process meant that readily available carbon was scarce in the short term, limiting the microbial ability to assimilate carbon, and consequently the carbon sequestration efficiency was lower [39,40].

The Lou soil we used in this experiment contains abundant calcium-rich minerals, and the mineral adsorption sites were not yet saturated. According to soil carbon saturation theory, this type of soil tends to fix recalcitrant organic matter more easily, forming a long-term stable soil carbon pool [25,26]. Notably, the C_mic_ enriched in the WS treatment belongs to the labile carbon fraction. Although its content was high in the short term, its structural stability is low, indicating that a small risk of re-mineralization and subsequent GHG emissions remains. Thus, there is a clear trade-off between short-term carbon sequestration efficiency and long-term carbon stabilisation potential for the two organic materials.

In summary, our findings clarify how soil carbon pools differentiate under the anaerobic conditions of the RSD system, revealing the mechanisms by which organic materials of different qualities regulate soil carbon fractions.

### 4.3. Net Climatic Trade-Offs Between Carbon Sequestration and GHG Emissions

Building on the observed differences in enzyme function and carbon fraction distribution, we used the NGWP index to integrate these two pathways into a single net climatic metric. To determine whether soil carbon sequestration and GHG emissions are synergistic or mutually offsetting under the RSD regime, we introduced the NGWP index. This index systematically examined the inherent trade-offs between soil carbon sequestration benefits and the greenhouse effect under anaerobic reducing conditions. The quantitative results for each treatment are summarised in Table 3.

The CK received no external organic carbon and remained continuously flooded and anaerobic throughout the experiment. Consequently, native SOC was progressively mineralized and decomposed, leading to an overall loss of the soil carbon pool. Interestingly, although the CK treatment had the lowest total GWP, accounting for the negative effect of soil carbon loss revealed that the entire system still maintained a positive net GHG load. This suggests that waterlogging and anaerobic conditions alone cannot achieve carbon sequestration in agricultural soils; instead, they disrupt the native carbon balance of the soil. This finding aligns with the conclusions of Zhu et al. (2013) [41] regarding anaerobic carbon turnover in greenhouse soils. In contrast, the two RSD treatments—WS and KB—compensated for the mineralization loss of native organic carbon through external carbon supply and ultimately achieved a soil carbon surplus. While both materials performed similarly in carbon sequestration capacity, with WS providing a slight edge in short-term carbon storage, addition of organic materials significantly increased the overall GHG load. Specifically, the total GWP of the WS treatment was markedly higher than that of the KB treatment. Even when accounting for the climate mitigation benefits of soil carbon sequestration, both organic-amended RSD treatments remained net sources of GHG emissions. In other words, the carbon sequestration gains from organic carbon input failed to offset the warming effect driven by sharp increases in methane emissions. This is a well-recognized trade-off of anaerobic organic amendment systems [8].

The marked difference in GHG emission profiles between the two treatments can be attributed primarily to methane. Methane was the key component driving the GWP divergence between the WS and KB treatments, contributing more than 99% of the total warming effect. The finding that CH_4_ accounted for over 99% of total GWP contrasts sharply with most field-based RSD studies, where N_2_O typically dominates the warming effect due to denitrification of residual nitrate [14,42]. This contrast arises from the combined effects of high temperature (45 °C) favouring thermophilic methanogens, the closed system limiting CH_4_ oxidation, and strongly reducing conditions suppressing N_2_O production [31]. The overwhelming dominance of CH_4_ in our system therefore represents a high-temperature, closed-system phenomenon rather than a direct reflection of field RSD practices. Wheat straw exhibits a low C/N ratio and is rich in easily decomposable organic matter, rendering it highly accessible to anaerobic microorganisms under the strongly reducing conditions of RSD. Consequently, this enhanced microbial assimilation efficiency and strengthened short-term soil carbon sequestration [15], while simultaneously supplying active substrates to methanogenic communities, thereby triggering substantial methane emissions. Kiwifruit branches, by contrast, have a higher C/N ratio and a larger proportion of lignified, recalcitrant components. Their organic matter decomposes at a slower pace, which effectively reduces the risk of concentrated methane release, resulting in a lower overall greenhouse warming load. Notably, both organic materials substantially reduced soil N_2_O emissions, with reductions exceeding 85% compared with the CK. Organic carbon input stimulated microbial nitrogen immobilisation and consumed soil nitrate-nitrogen, thereby suppressing denitrification. This pattern of nitrogen transformation in anaerobic soils aligns with several similar studies [32]. However, because the baseline N_2_O emissions across all treatments were already very low, this variable was not the main driver of the differences in net climate effects between the two treatments.

In summary, organic materials with different C/N ratios exhibit clear functional trade-offs within the RSD management system. This C/N-driven carbon-climate trade-off is conceptually summarized in graphical abstract. Wheat straw, with its low C/N ratio, is more suitable for short-term soil fertility improvement and rapid enhancement of soil carbon storage, yet it carries the environmental risk of elevated methane emissions. Kiwifruit branches, with their higher C/N ratio, offer only modest short-term carbon gains while effectively mitigating overall GHG emissions, aligning more closely with low-carbon agricultural objectives. Therefore, the substrate structure and decomposition characteristics of organic amendments directly determine the pathway of soil carbon turnover and the pattern of GHG emissions under anaerobic conditions [27]. This underlies the marked divergence in carbon sequestration and climate effects observed between the WS and KB treatments in this study. The NGWP analysis above also answers whether the observed carbon gain can offset the warming from CH_4_. Under our experimental conditions, the answer is negative: even with the C-seq benefit deducted, both WS and KB remained net GHG sources (Table 3). This indicates that the environmental benefits of RSD were largely negated by elevated CH_4_ emissions under the high-temperature anaerobic regime used here. However, as noted in Section 4.1, the 45 °C incubation may overestimate field CH_4_ fluxes; thus, the net climate benefit of RSD in practice remains an open question that warrants field-based verification. Figure 4 integrates CSE with CH_4_-GWP and NGWP, highlighting the contrasting trade-offs of the two amendments. WS achieves higher CSE but at a substantially greater methane cost, resulting in a larger NGWP. KB delivers more moderate CSE but imposes a markedly lower methane burden and NGWP. This divergence stems from substrate C/N ratio-driven differences in decomposition kinetics and carbon partitioning between methanogenic and non-methanogenic pathways. Practically, WS is preferable for rapid SOC build-up, whereas KB better serves the goal of minimizing net climate warming in RSD management.

The comparison between WS and KB also raises the question of whether blending the two materials could achieve a more favourable balance. Given that WS promotes C_mic_ accumulation while KB favours C_min_ formation, a mixture might theoretically combine rapid carbon build-up with reduced methanogenic risk. However, the present study did not test blended amendments, and the interactive effects between labile and recalcitrant fractions on methanogenesis remain unknown. Similarly, the effect of amendment application rate on GHG trade-offs was not examined, as only a single rate (2% *w*/*w*) was used. Future studies should systematically vary both the mixing ratio and the total addition rate to identify optimal RSD amendment strategies.

### 4.4. Limitations and Future Research Directions

This study has several limitations. First, the incubation conditions were set to a constant temperature of 45 °C, continuous waterlogging, and a nitrogen-purged closed environment, which differ markedly from actual field conditions. In the field, environmental variables such as diurnal temperature fluctuations, localized aerobic microsites within the soil, and root exudate inputs are present. These controlled conditions may have amplified methane emissions and short-term carbon sequestration effects to some extent. Consequently, the conclusions of this study cannot be directly extrapolated to in situ agricultural field conditions [8]. Second, this study evaluated only a greenhouse Lou soil collected from the Guanzhong region of Shaanxi Province. Whether the observed regulation patterns of these organic materials apply to soils with different textures or baseline fertility levels remains to be verified. Third, the NGWP calculation assumes that the measured SOC increase can be treated as a CO_2_ offset. However, since the 30-day incubation only captures short-term carbon retention, this offset should not be equated with permanent sequestration. The NGWP values reported here are therefore best viewed as time-bound estimates that reflect the net warming potential within the experimental period rather than a long-term net climate benefit. In addition, we did not monitor redox potential (Eh) or volatile fatty acids (VFAs) during incubation, nor did we track nutrient (e.g., P, K) dynamics over time. Moreover, the correlative nature of our enzyme–gas data precludes definitive causal inference. We also did not examine operational measures that might reduce CH_4_ emissions, such as adjusting the application rate, shortening the flooding period, or introducing post-treatment aeration; only one rate (2% *w*/*w*) was tested, and WS and KB were used as separate amendments without blending. More importantly, our two-material comparison does not allow us to attribute the observed differences solely to C/N ratio, since WS and KB also differ in lignocellulose content and decomposability. Disentangling the specific role of C/N from these confounding factors would require a gradient experiment using a broader range of materials or blended mixtures. These questions remain open and merit further investigation.

To address these limitations, future research could focus on the following aspects. First, long-term field experiments should be established to monitor GHG fluxes in situ using static chambers, while simultaneously tracking the interannual dynamics of soil carbon fractions. Second, metagenomic approaches combined with isotope labelling techniques could be employed to identify key microbial taxa involved in regulating carbon decomposition, methanogenesis, and denitrification. Third, co-application experiments using organic materials with contrasting (high vs. low) C/N ratios are needed to determine the optimal mixture that balances high carbon sequestration efficiency with low GHG emissions.

## 5. Conclusions

Based on a 30-day anaerobic incubation at 45 °C, this study yielded four main findings regarding the role of organic amendment C/N ratio in regulating soil carbon turnover, enzyme activities, and GHG emissions under RSD. These results were obtained under controlled laboratory conditions, and the elevated temperature may have enhanced methanogenic activity, potentially overestimating CH_4_ emissions relative to field scenarios. Field-scale validation over longer time frames is needed before extrapolating these findings to agronomic practice.

(1) The C/N ratio of organic materials served as the primary control on GHG emission patterns. Compared with CK, WS increased cumulative CO_2_ by 21-fold (to 1111.81 mg kg^−1^) and CH_4_ by 424.47 mg kg^−1^, whereas KB produced 18.7-fold higher CO_2_ (989.55 mg kg^−1^) and 24% less CH_4_ (322.78 mg kg^−1^) than WS. Both amendments suppressed N_2_O by >85%, with CK emitting 6–7 times more N_2_O than the amended treatments. These results confirm that substrate C/N ratio drives distinct GHG trade-offs during RSD.
(2) Adding exogenous organic carbon expanded soil carbon pools and altered carbon fraction distribution. WS and KB both increased TC, SOC, DOC, MBC, C_mic_, and C_min_ relative to CK. Notably, WS favoured C_mic_ accumulation (17.9% higher than KB) and stimulated hydrolytic enzyme activities (βG, CBH), whereas KB promoted C_min_ formation (16.2% higher than WS) and showed higher XYL activity at day 5 (69.8% above WS). These contrasting outcomes arose from divergent retention pathways tied to substrate decomposability.
(3) The C/N ratio influenced carbon sequestration efficiency. WS achieved a CSE of 25.79%, significantly higher than KB (20.11%), while the unamended CK showed net SOC mineralisation (−8.20%). Thus, low-C/N WS offered faster short-term carbon build-up, whereas high-C/N KB favoured slower but more stable accumulation.
(4) NGWP analysis exposed a fundamental trade-off. Both WS and KB remained net GHG sources, but KB showed a substantially lower NGWP (611.60 t CO_2_-eq ha^−1^) than WS (798.19 t CO_2_-eq ha^−1^). High-C/N KB thus delivered a smaller carbon gain but a much lower net warming impact, making it more compatible with low-carbon agricultural goals.

It should be borne in mind that these conclusions derive from a short-term laboratory experiment with constant temperature and strictly anaerobic conditions. While the 45 °C setting approximates summer soil temperatures in RSD-treated fields, it may favour thermophilic methanogens and produce CH_4_ fluxes that are not representative of fluctuating field environments. Moreover, the incubation system excluded plant roots, drying–rewetting events, and the spatial heterogeneity typical of cultivated soils. Consequently, the GWP and NGWP estimates reported here are best viewed as upper-bound values. Longer-term field studies are necessary to determine whether the observed SOC gains persist and how net climate effects manifest under real farming conditions.

Overall, the C/N ratio of organic materials incorporated during RSD shapes microbial metabolism, enzyme efficiency, and carbon transformation pathways, thereby influencing soil carbon cycling, GHG emissions, and net climate outcome. Where rapid carbon build-up is the priority, low-C/N wheat straw is the preferred choice; where reducing the net climatic footprint matters more, high-C/N kiwifruit branches are better suited. This study offers both theoretical insight and practical guidance for selecting organic materials, regulating carbon–nitrogen cycling, and mitigating GHG emissions in RSD-based remediation of degraded soils.

## Figures and Tables

**Table 1:** Basic properties of the soil used.

Initial Soil Properties	SOC (g kg^−1^)	DOC (mg kg^−1^)	MBC (mg kg^−1^)	NO_3_^−^-N (mg kg^−1^)	NH_4_^+^-N (mg kg^−1^)	pH	EC (μs cm^−1^)
Initial	8.62	119.40	166.99	44.19	7.27	8.23	367.7

SOC, soil organic carbon; DOC, dissolved organic carbon; MBC, microbial biomass carbon; EC, electrical conductivity.

**Table 2:** Basic properties of plant residues used.

Materials	TC (%)	TN (g kg^−1^)	TP (g kg^−1^)	TK (g kg^−1^)	C/N
Wheat straw	41.1	7.4	5.2	29.3	55.5
Kiwifruit branches	56.5	5.1	10.2	16.2	110.8

TC, total carbon; TN, total nitrogen; TP, total phosphorus; TK, total potassium; C/N, carbon-to-nitrogen ratio.

**Table 3:** Climatic effects of GHG emissions and carbon sequestration.

Treatment	GWP (t CO_2_-eq ha^−1^)			Total GWP	C-seq	NGWP
	CO_2_	CH_4_	N_2_O	(t CO_2_-eq ha^−1^)	(t CO_2_-eq ha^−1^)	(t CO_2_-eq ha^−1^)
CK	0.14 c	0.031 c	0.276 a	0.45 c	−2.78 b	3.23 c
WS	2.75 a	820.72 a	0.044 b	823.52 a	25.33 a	798.19 a
KB	2.37 b	633.82 b	0.043 b	636.23 b	24.63 a	611.60 b

Notes: Lowercase letters denote significant differences among treatments (*p* < 0.05). C-seq = CO_2_ equivalent of soil carbon sequestration; NGWP = net global warming potential. Calculations used a soil bulk density of 1.418 g cm^−3^ for the 0–20 cm layer, with a conversion factor of 3.67 t CO_2_ per ton of organic carbon. A negative C-seq value indicates a net loss of SOC. Therefore, the NGWP exceeds the total GWP for this treatment. The C-seq offset represents the CO_2_ equivalent of the net SOC increase measured after 30 days of incubation, and should not be interpreted as permanent carbon sequestration.

## Data Availability

The original contributions presented in this study are included in the article and Supplementary Materials. Further inquiries can be directed to the corresponding author.

## Supplementary Materials

The following supporting information can be downloaded at: https://www.mdpi.com/article/10.3390/metabo16090653/s1, Figure S1. Effects of different organic materials on soil pH and EC.
