# Supplementary material for: Organic Amendment Quality Regulates Greenhouse Gas Trade-Offs and Short-Term Carbon Retention During Reductive Soil Disinfestation
Source: Metabolites. 2026 Sep 6;16(9):653. doi: 10.3390/metabo16090653 (PMC13609707; doi:10.3390/metabo16090653)
Supplement: Supplementary file 1 [file metabolites-16-00653-s001.zip › metabolites-4487054-supplementary.pdf]

metabolites

MDPI

Supplementary Material

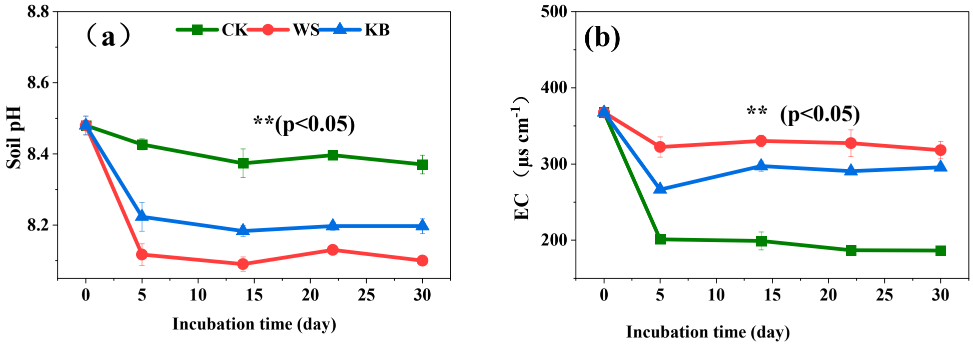

Figure S1. Effects of different organic materials on soil pH and EC.
